# Injectable Lyophilized Chitosan-Thrombin-Platelet-Rich Plasma (CS-FIIa-PRP) Implant to Promote Tissue Regeneration: In Vitro and Ex Vivo Solidification Properties

**DOI:** 10.3390/polym15132919

**Published:** 2023-06-30

**Authors:** Fiona Milano, Anik Chevrier, Gregory De Crescenzo, Marc Lavertu

**Affiliations:** 1 Biomedical Engineering Institute, Polytechnique Montreal, Montréal, QC H3T 1J4, Canada; 2 Chemical Engineering Department, Polytechnique Montreal, Montréal, QC H3T 1J4, Canada

**Keywords:** chitosan, thrombin, platelet-rich plasma, injectable implant, tissue regeneration, solidification properties

## Abstract

Freeze-dried chitosan formulations solubilized in platelet-rich plasma (PRP) are currently evaluated as injectable implants with the potential for augmenting the standard of care for tissue repair in different orthopedic conditions. The present study aimed to shorten the solidification time of such implants, leading to an easier application and a facilitated solidification in a wet environment, which were direct demands from orthopedic surgeons. The addition of thrombin to the formulation before lyophilization was explored. The challenge was to find a formulation that coagulated fast enough to be applied in a wet environment but not too fast, which would make handling/injection difficult. Four thrombin concentrations were analyzed (0.0, 0.25, 0.5, and 1.0 NIH/mL) in vitro (using thromboelastography, rheology, indentation, syringe injectability, and thrombin activity tests) as well as ex vivo (by assessing the implant’s adherence to tendon tissue in a wet environment). The biomaterial containing 0.5 NIH/mL of thrombin significantly increased the coagulation speed while being easy to handle up to 6 min after solubilization. Furthermore, the adherence of the biomaterial to tendon tissues was impacted by the biomaterial-tendon contact duration and increased faster when thrombin was present. These results suggest that our biomaterial has great potential for use in regenerative medicine applications.

## 1. Introduction

Our laboratory developed a freeze-dried (FD) formulation of chitosan (CS) intended to be solubilized in platelet-rich plasma (PRP) to form injectable implants that coagulate in situ [1]. This biomaterial is intended to be used as a surgical adjuvant in diverse applications in orthopedic regenerative medicine.

Chitosan (CS) is a cationic linear polymer consisting of monomers of N-acetyl glucosamine and glucosamine monomers, which is usually obtained through alkaline deacetylation of chitin [2]. CS has been found to exhibit a greater affinity for cells and growth factors than other natural polymers, most likely because of its ability to mediate attractive electrostatic interactions [3]. CS is biodegradable and biocompatible [2,4], and it has numerous applications in the biomedical field [5]. These applications include drug delivery, gene therapy, vaccines, tissue engineering [6], and wound healing [7]. CS’s interesting properties in wound healing include its antimicrobial, hemostatic, and analgesic character [8]. Moreover, CS influences the coagulation cascade by mediating hemagglutination and promoting platelet activity [6]. CS molecular weight and degree of deacetylation (DDA, the fraction of glucosamine monomers) impact the innate immune responses [6], and chitosan is considered an excellent candidate as a mucosal adjuvant [9]. Overall, the unique properties of CS make it an attractive polymer for a variety of biomedical applications.

PRP is a plasma fraction containing a platelet concentration several times higher than that of whole blood (WB) [10]. Upon activation, these platelets release several growth factors [11], which are believed to promote tissue repair by inducing cell proliferation, cell differentiation, and angiogenesis at the injury site [12]. However, the literature on the efficacy of PRP in improving tissue repair has yielded inconsistent results, and there is currently insufficient clinical evidence to support its use [13]. These inconsistencies may arise from the lack of standardization of platelet separation techniques, the variability in formulations of platelet separation techniques, the variability in formulations of PRP used, as well as the poor stability and residency of PRP in vivo [13,14]. 

Using PRP instead of WB to rehydrate FD-CS formulations is expected to enhance the bioactivity of the resulting hybrid implants and improve tissue repair outcomes [1]. Our previous research has demonstrated that CS increases the release of platelet-derived growth factor, prolongs the release of platelet-derived growth factor, prolongs the residence time, and enhances the bioactivity of PRP in vivo while inhibiting the platelet-mediated clot retraction observed with the use of PRP alone [15]. Animal studies have confirmed that CS-PRP implants are biodegradable, biocompatible, and effective in augmenting rotator cuff, cartilage, and meniscus repair (increase cell recruitment, vascularization, remodeling, and tissue repair) in both small and large animal models [14,16,17]. Moreover, CS-PRP implants possess important characteristics for soft tissue repair, such as injectability, stickiness, paste-like handling properties, and the ability to solidify in situ.

However, mixing FD-CS with PRP results in a hybrid clot implant that solidifies slowly, especially for human PRP. When applied directly after mixing, the wound’s or defect’s wet environment can hinder the biomaterial from adhering to the site of injection. In an ongoing rotator cuff multicenter, prospective, randomized clinical trial (ClinicalTrials.gov, NCT number NCT05333211, Sponsor: ChitogenX Inc., Kirkland, QC, Canada), the biomaterial is required to be incubated in a syringe for 30–45 min before reaching a viscosity suitable for injection at the repaired site. A more rapid and controlled implant solidification would facilitate its handling, its administration, and ultimately its clinical implementation. 

The objective of this study was to develop a novel formulation of FD-CS with accelerated solidification. We hypothesized that the solidification of CS-PRP implants can be accelerated by incorporating thrombin (FIIa), as previously reported for CS-glycerol phosphate/blood implants [18]. Our selection of this coagulation factor was based on its well-known ability to promote the repair process as well as its established use in other clinical contexts [19,20]. A second hypothesis was that thrombin could be added prior to FD to create a stable CS-FIIa-lyophilized formulation. Adding thrombin prior to FD (1) ensures that thrombin is evenly dispersed in the biomaterial upon rehydration in PRP, and (2) simplifies the procedure compared to in situ mixing of the CS-PRP implant with a thrombin formulation/solution. However, this approach presents a challenge: the formulation should coagulate fast enough to be applied in a wet environment upon mixing, but not so quickly that it becomes difficult, if not impossible, to handle/inject. To achieve this balance, CS-PRP biomaterials containing thrombin concentrations of up to 1.0 NIH/mL were studied in vitro (using thromboelastography, rheology, indentation, syringe injectability, and thrombin activity tests) as well as ex vivo (by assessing the implant’s adherence to tendon tissue in a wet environment). 

## 2. Materials and Methods

### 2.1. Preparation of Freeze-Dried Chitosan Formulations

The FD-CS formulations preparation were based on the method developed by Chevrier et al. (2018) [1]. Raw chitosan (Primex, Siglufjordur, Iceland, Product N°43030) underwent heterogeneous alkaline deacetylation to reach a DDA of 82 ± 2 % (mean value ± SD for 3 batches, measured by nuclear magnetic resonance spectroscopy [21]). It was then depolymerized with nitrous acid to reach a molar mass ($M_{n}$) of 39.3 ± 0.4 kDa (mean value ± SD for 3 batches, measured by size-exclusion chromatography/multi-angle laser light scattering [22]). CS was dissolved in HCl overnight at room temperature to obtain a solution containing 1% (*w*/*v*) CS. The concentration of this HCl solution was adjusted to reach 60% protonation of the CS amino groups. Calcium chloride (Sigma-Aldrich, St. Louis, MO, USA, Product N°C-5670) was added as a solidifying agent to reach a final concentration of 42.2 mM, and then trehalose (Sigma-Aldrich, St. Louis, MO, USA, Product N°T-0167) was added as a lyoprotectant to reach a final 1% (*w*/*v*) content. The resulting solution was passed through 0.45 μm filters and dispensed in glass vials. The pH of the chitosan solutions was 6.35 ± 0.16 and the osmolality was 199 ± 21 (mean value ± SD for 9 batches).

For formulations containing thrombin, thrombin diluted in a 0.1% (*w*/*v*) BSA solution was added to each glass vial to reach a thrombin activity of 0.25 NIH/mL, 0.50 NIH/mL, or 1.0 NIH/mL and a final BSA concentration of 0.01% (*w*/*v*). All vials were then freeze-dried through the following cycle using a Millrock freeze-dryer (Laboratory Series LD85S3, Millrock Technology, Kingston, NY, USA): (1) step freezing (to 20 °C then isothermal for 15 min, to 5 °C then isothermal for 30 min, to −5 °C then isothermal for 30 min) then ramp freezing to −40 °C at −1 °C/min, then isothermal for 2 h; (2) ramp heating at 0.5 °C/min up to 8 °C at 76 mTorr, then isothermal for 650 min; (3) ramp heating at 0.15 °C/min until 40 °C at 76 mTorr, then isothermal for 6 h. A less aggressive cycle was initially used, heating up to only 25 °C during step 3 (secondary drying), but for 10 h. However, no differences were observed in the resulting FD-CS formulations, and the cycle heating up to 40 °C was preferred for the rest of the study. Of note, some of the data used in Section 3.1 (thromboelastography results) were obtained from the less aggressive FD cycle.

### 2.2. Isolation of Platelet-Rich Plasma

Commercially sterile citrated sheep blood (Cedarlane, Burlington, ON, Canada, Product N°CL2581-500C) was used to isolate the leucocyte-rich PRP. Briefly, whole blood was distributed in 10 mL aliquots in 15 mL Falcon tubes, then centrifuged at 800× *g* for 20 min (unforced deceleration). The supernatant, buffy coat, and first 1-2 mm of erythrocytes were collected in new 15-mL Falcon tubes, and a second centrifugation was then performed at 600× *g* for 10 min (unforced deceleration). 

### 2.3. Rehydration of the CS-FD Formulation

Freeze-dried CS cakes were solubilized in PRP by vigorous manual shaking for 30 s. The cakes were made from various volumes of CS solutions, depending on the final amount of biomaterial needed (1 mL, 3 mL, 5 mL, 6 mL, or 8 mL), and rehydrated in the same volume of PRP.

### 2.4. Assessment of Clotting Properties of CS-PRP Formulations by Thromboelastography

After dissolution of the CS cakes in PRP, 360 μL of each formulation was immediately loaded in a thromboelastograph (TEG) cup using a 1 mL syringe and an 18 G needle (allowing the viscous formulation to be easily retrieved from the vial). TEG tracings were recorded for 1 h, and three parameters were used to compare the samples: (1) The clot reaction time, R, is the time in minutes to reach a 2 mm divergence between the two branches of the TEG curve and is indicative of the time needed for the coagulation process to start. (2) The maximal amplitude, MA, is the maximal distance reached between the two branches of the TEG curve (in mm) and is indicative of the clot strength. (3) The K-value, which is the duration between R and the time when a 20 mm divergence between the two branches of the TEG curve is reached and is indicative of the coagulation speed. Two PRP solutions were used as controls. The first consisted of PRP recalcified in 42.2 mM of calcium chloride. The second consisted of PRP recalcified in 42.2 mM of calcium chloride and 0.5 NIH/mL of thrombin. A TEG Model 5000 (Haemoscope Corp., Niles, IL, USA) was used for all samples.

### 2.5. Assessment of Clotting Properties of CS-PRP Formulations through Rheology Measurements

Clotting properties were measured using a Physica MCR 501 rheometer (Anton Paar, Graz, Austria). Concentric cylinders with rough geometry were used for measurements (CC17/T200/SS/p, cup diameter of 18.08 mm, and bob diameter of 16.66 mm). After mixing the CS-FD formulation (8 mL) with PRP, 5 mL of the formulation was placed in the cup. Rheological properties were measured during a time sweep over the course of either 1 h or 15 min at a fixed 1% strain and a fixed 5 rad/s frequency. The choice of these parameters is explained and justified in Appendix A. All measurements were performed at 37 °C.

### 2.6. Assessment of the Force Required to Eject the Biomaterial

The protocol used was implemented on the multi-axis mechanical tester Mach-1, Model V500css (Biomomentum Inc., Laval, QC, Canada), with a 25 kg uniaxial load cell and a flat circular indenter with a diameter of 30 mm. 6 mL of each sample were placed in a 10 mL syringe with an 18 G × 3.50 IN quincke spinal needle (BD Product N°405184) right after rehydration of the FD-CS formulation in PRP. The syringe was then fixed vertically below the indenter through a custom system. Starting right after the syringe’s fixation, 0.5 mL of the biomaterial was pushed every 2 min, 10 times, at a speed of 1 mm/s (resulting in 3 s of movement). Force data were saved at a frequency of 100 Hz for the whole duration of the test.

### 2.7. Assessment of Hybrid-Clot Mechanical Properties through Indentation

The indentation protocol used was implemented on the multi-axis mechanical tester Mach-1, Model V500css (Biomomentum Inc., Laval, QC, Canada) with a 150 g uniaxial load cell and a spherical indenter with a radius of 1.5 mm (<25% of the sample height—~8 mm in our case—as previously recommended in the literature [23,24]). 0.3 mL of each sample was placed in a 96-well microplate and allowed to coagulate for 24 h at room temperature. The bottom of the microplate was first probed in three empty wells to fit a plane and compute the position of the bottom in each well. For each sample, the surface of the biomaterial was then found by lowering the probe at a speed of 0.05 mm/s until a force of 0.1 gf was detected. A single indentation of 0.7 mm (<10% of the total sample height, as previously recommended in the literature [24,25] to prevent the influence of the sample height) was then performed at a velocity of 0.07 mm/s. Force data were saved at a frequency of 100 Hz during the indentation and the relaxation that followed, until the relaxation rate reached 1 gf/min (slope measured for 10 s). Sample stiffness and force at equilibrium were computed for each sample. 

### 2.8. Assessment of Hybrid-Clot Homogeneity through Histology and MASQH Algorithm

After rehydration of CS-FD cakes in PRP, the formulations were placed in glass test tubes (250 μL) at 37 °C and solidified for 1 h. The resulting clots were fixed in 10% neutral buffered formalin (NBF) before being dehydrated, cleared, and paraffin embedded. They were then sectioned into 5 μ m-thick slices, dewaxed, and stained. Cibacron Brilliant Red (Glentham Life Sciences, Product N°GT9393) and Iron-Hematoxylin (Sigma, Products N°HT107, and N°HT109) were used to color chitosan, red, and white blood cells, as per the protocol described in Rossomacha et al. [26]. The colored slices were digitally scanned (Hamamatsu Nanozoomer RS) at 10× and 40×. The homogeneity of the chitosan distribution in the hybrid clot was evaluated using the MASQH algorithm [27].

### 2.9. Assessment of CS-PRP Formulations Adhesion to Tendon Tissues Using an Ex Vivo Test

The objective of this test was to determine whether the biomaterial could adhere to wet tissues. Ex vivo evaluation of the CS-PRP formulations adhesion to tendon tissues was performed using bovine tendons obtained from a local butcher shop. Prior to testing, tendons were defrosted and heated to 37 °C in a 0.9% NaCl bath, a frequently used fluid during shoulder arthroscopic surgeries [28]. Right after mixing a CS-FD cake with PRP, the formulation was retrieved from its vial with a 10 mL syringe and an 18 G needle (Sigma, Product N°305180), and a waiting time varying between 0 and 7 min was observed to mimic the time taken by the surgeon between mixing the biomaterial and its use. Next, 3 mL of the biomaterial was delivered to the tendon, and a second waiting time varying between 0 and 7 min was observed. During the surgery, after suturing the tendon to the bone, the irrigation fluid is stopped, and then the surgical site is dried with a combination of fluid aspiration and sterile cotton swabs. The biomaterial is then delivered at the tendon-bone interface and on the tendon before closing the articulation. The second waiting time, therefore, mimics the conditions after the biomaterial application and the end of the surgery, as the physiological fluid does not return instantly to the articulation. After this second waiting time, the tendon and adhered biomaterial were submerged in a 1 L 0.9% NaCl bath and twisted in the fluid for 1 min to simulate the presence of physiological fluids and movement of the shoulder. The quantity of biomaterial remaining on the tendon was then visually observed (qualitative evaluation). The color of the 0.9% NaCl bath was quantitatively evaluated through image analysis: a photograph of the bath was taken, and the weight of its red channel was computed using the ImageJ software [29]. 

### 2.10. Assessment of Thrombin Activity in Freeze-Dried Chitosan Formulations

The activity of thrombin in the CS formulations was quantified before FD and in CS-FD cakes using a fluorometric thrombin activity assay kit (Abcam, ab197006). FD samples were rehydrated in water instead of PRP. All samples were diluted at 1/2 and 1/5 in assay buffer. As per Abcam protocol, six standards were used to construct a standard curve, with concentrations ranging from 0.26 NIH/mL to 1.3 NIH/mL. Fluorescence was measured at Ex/Em = 350/450 at 37 °C in a kinetic mode every 2 min for 60 min using an Infinite M200 reader (Tecan Trading AG, Männedorf, Switzerland).

### 2.11. Statistical Analysis

To investigate whether thrombin accelerates coagulation significantly, the TEG results were subjected to statistical analysis using the Mann-Whitney U test. Similarly, the same test was performed on the TEG results to determine if there was any alteration in the biomaterial activity due to the storage time. Differences were considered significant for *p*-values < 0.05. The figure’s caption accompanying each result contains information on the number of independent experiments conducted (*N*) as well as the number of replicates for each sample in these experiments (*n*). 

## 3. Results and Discussion

### 3.1. Chitosan Freeze-Dried with Thrombin Easily Solubilizes in PRP, Exhibits Thrombin Activity, and Maintains Stability for at Least 2 Months at Room Temperature

**Figure 1:**
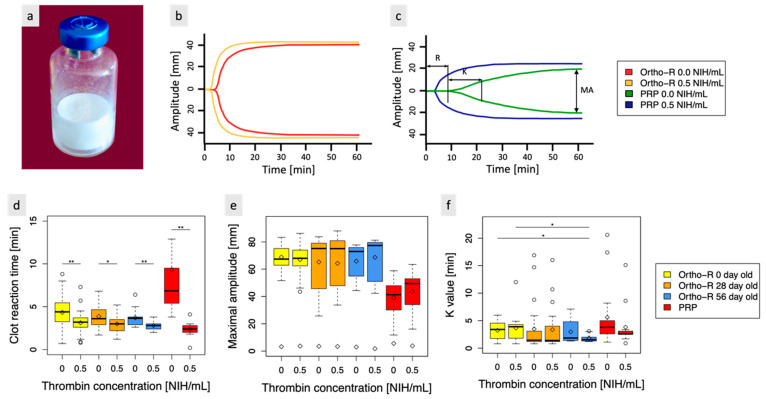
(**a**) The biomaterial, which is a lyophilized formulation of chitosan, is designed to be mixed with platelet-rich plasma (PRP) to create a hybrid biomaterial that coagulates in situ. To ensure rapid coagulation in situ during arthroscopic surgery, thrombin was added to the existing formulation. (**b**) Typical thromboelastograph curves obtained for biomaterials with and without thrombin. (**c**) Typical thromboelastograph curves obtained for PRP with and without thrombin. (**d**) clot reaction time (R) (time needed to reach an amplitude of 2 mm), (**e**) clot maximal amplitude (MA) (indicative of clot strength), and (**f**) K value (time needed after R to reach an amplitude of 20 mm). * indicates a *p*-value < 0.05, ** indicates a *p*-value < 0.01, and ◊ indicates the mean value. Note that statistically significant differences were also observed for the R values between samples with and without thrombin issued from different timepoints but are not indicated in the figure for clarity’s sake. For the biomaterial with and without thrombin at *t* = 0, *N* = 7 and *n* = 3, at *t* = 1 month, *N* = 6 and *n* = 3, and at *t* = 2 months, *N* = 2 and *n* = 3. For PRP controls with and without thrombin, *N* = 6 and *n* = 2.

Freeze-dried chitosan formulations (Figure 1a) were slightly retracted from the vial walls for all thrombin concentrations (this phenomenon was expected and previously observed by our research group [1]). They rapidly solubilized in PRP without any apparent influence on the thrombin concentration. 

The pH of whole blood was 7.69 ± 0.13, and the pH of PRP was 7.97 ± 0.18 (mean value ± SD for 12 batches). The osmolality of whole blood was 316 ± 3 and the osmolality of PRP was 317 ± 2 (mean value ± SD for 8 batches). The pH of the CS-PRP formulation, with or without thrombin, was 7.20 ± 0.16 (mean value ± SD for 15 batches).

An activity loss of ~20–25% was observed after lyophilization for samples containing 0.5 NIH/mL of thrombin, which was the only formulation assessed using the thrombin activity kit. In the remainder of the manuscript, the thrombin activity mentioned will refer to the activity before lyophilization (i.e., not taking into account the ~25% activity loss). The 0.5 NIH/mL formulation was also tested for thrombin activity with and without CS before freeze-drying, and no difference was observed. This suggests that the interaction between thrombin and CS is weak or nonexistent, as previously reported in [30,31]. This expected result is likely due to the cationic character of chitosan and thrombin at the pH of the solution. The histology of CS-FIIa-PRP implants confirmed that CS was evenly distributed among blood components in the presence of thrombin. A MASQH homogeneity score of 0.92 ± 0.03 was computed for CS-PRP implants and of 0.93 ± 0.02 for CS-FIIa-PRP implants (mean value ± SD for 5 samples in each case). 

A stability study was conducted for a period of two months, and thrombin activity was assessed at three specific time points (t = 0, 28, and 56 days) using TEG. All the samples were stored at room temperature (RT). Some FDA-approved biomaterials containing thrombin were stored at temperatures below −18 °C (they contain solubilized human thrombin for topical use, e.g., Evithrom^®^, Tisseel^®^, and Evicel^®^ pre-filled syringes), and others were stored at RT (they contain lyophilized human thrombin powder, e.g., Recothrom^®^, and Tisseel^®^ kit).

This study investigated the stability of biomaterials without thrombin (controls) and of biomaterials containing 0.5 NIH/mL of thrombin. Additionally, freshly extracted PRP controls without thrombin or with 0.5 NIH/mL of thrombin were also included. Examples of typical TEG curves are presented in Figure 1b for biomaterial samples and in Figure 1c for PRP samples. No other thrombin concentration was tested, as 0.5 NIH/mL was previously identified as the preferred concentration (this choice is justified in Section 3.2 below). The results showed that thrombin had a statistically significant impact on the biomaterial’s clot reaction time compared to the biomaterial without thrombin (control) over a period of up to 56 days (*p*-value < 0.01 for t = 0 and t = 2 months and *p*-value < 0.05 for t = 1 month, as shown in Figure 1d). The average clot reaction time (R) for the control biomaterial was 4.04 min, while biomaterial with 0.5 NIH/mL of thrombin had an average R of 3.02 min over the three time points. No significant differences in clot reaction time were observed between control samples at each time point, which was expected as the biomaterial is already known to be stable for at least three years (unpublished data). Furthermore, no significant differences in clot reaction time were observed between samples with thrombin at each time-point, suggesting that no activity loss occurred during this 2-month period. No statistically significant differences were observed for the K-value between samples with and without thrombin for all three-time points (Figure 1e). Significant differences were, however, observed between samples with thrombin after two months and both samples observed at time 0 (*p*-value < 0.05). This could be explained by the small sample size (N = 2 and n = 3) for the two-month samples. Further studies are needed to confirm the observed trend. K had a mean duration of 3.45 min for control samples and 3.11 min for samples with 0.5 NIH/mL of thrombin across the three time points. Notably, MA had a consistent value of approximately 67 mm at all time points, irrespective of the thrombin concentration. This indicates that the addition of thrombin did not affect the strength of the final clot (no significant differences were observed, as shown in Figure 1f), as expected. 

Compared to PRP controls, biomaterial samples (with or without thrombin) had a smaller R, a smaller K, and a larger MA (*p*-value < 0.01). These findings are consistent with previous research conducted by Chevrier et al. [1], who also demonstrated that the addition of CS to blood samples enhanced clot strength and led to faster clot initiation. Furthermore, the addition of thrombin to PRP resulted in a significant decrease in clot reaction time but did not have a significant impact on K and MA. 

Long-term stability studies at −20 °C, 4 °C, and RT using thrombin activity quantification kits are ongoing.

Visual observation of the samples coagulation in plastic weighing boats confirmed that the coagulation process is faster when thrombin is present. Although the difference in coagulation time may appear moderate when examining the TEG results, it is significant when the handling properties of the samples are assessed. Supplementary Materials Video S1 includes videos demonstrating this phenomenon. 

### 3.2. Biomaterial Containing 0.5 NIH/mL of Thrombin Is Easily Dispensed through an 18-Gauge Needle and 10-cc Syringe up to Six Min after Rehydration of the Lyophilized Formulation in PRP

For every thrombin concentration, the force needed to eject the biomaterial out of a 10-cc syringe equipped with an 18-gauge needle increases over time, as expected since the coagulation process is ongoing in the syringe. After a few minutes (sooner for higher thrombin concentrations), the force required to eject the biomaterial reaches a maximum before dropping and/or stabilizing around 25 N.

**Figure 2:**
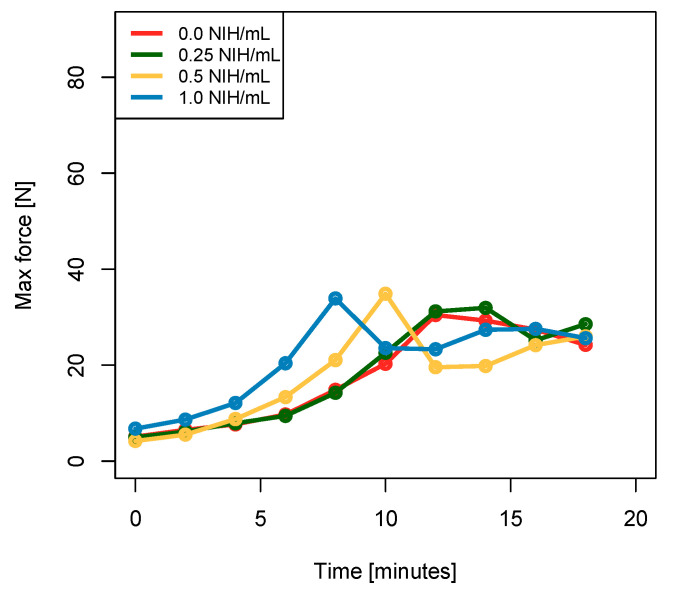
Force (lines indicate mean values and envelopes indicate min and max values) necessary to push 500 μL of the biomaterial out of a 10-cc syringe equipped with a spinal 18-gauge needle every two minutes, 20 times (*N* = 6 and *n* = 2). The 0.5 NIH/mL solution allows for easy ejection of the biomaterial as the force required for biomaterial ejection remains below 20 N for at least 6 min. This makes it easier to manipulate than the 1.0 NIH/mL solution, for which a force exceeding 20 N is needed for biomaterial ejection after 4–5 min.

We believe that this drop occurs when the applied force breaks the clot’s structure being formed (around 30–40 N on average, but reached values as high as 70–95 N, as shown in Figure 2). Once the structure is broken, the biomaterial becomes easier to eject, but the coagulation process is likely impaired, resulting in altered mechanical properties (as previously observed for PRP after stress [32] or for fibrin after a stretch or biaxial confinement [33,34]).

Even before reaching this maximal force, the high force required to eject the biomaterial might not allow the surgeon to perform precise movements, preventing him from precisely deliver/inject the biomaterial as expected. Indeed, a study by R. Watt et al. recommends targeting no more than 20 N of injection force to guarantee easy expulsion of syringe contents [35]. A force exceeding 20 N was observed as early as the 8th minute for controls and samples with 0.25 NIH/mL of thrombin, on the 6th minute for samples with 0.5 NIH/mL of thrombin, and between the 4th and 5th minutes for samples with 1.0 NIH/mL of thrombin, as shown in Figure 2. Based on this analysis, the 0.5 NIH/mL sample was identified as the most promising as it allows a significant increase in coagulation rate while preserving adequate handling properties for at least 6 min, providing enough time for the surgeon to use/deliver the biomaterial after its reconstitution.

### 3.3. Rheometry Reveals That Thrombin Concentration Has a Significant Impact on Storage and Loss Modulus, as Well as on the Time to Gelation Point

**Figure 3:**
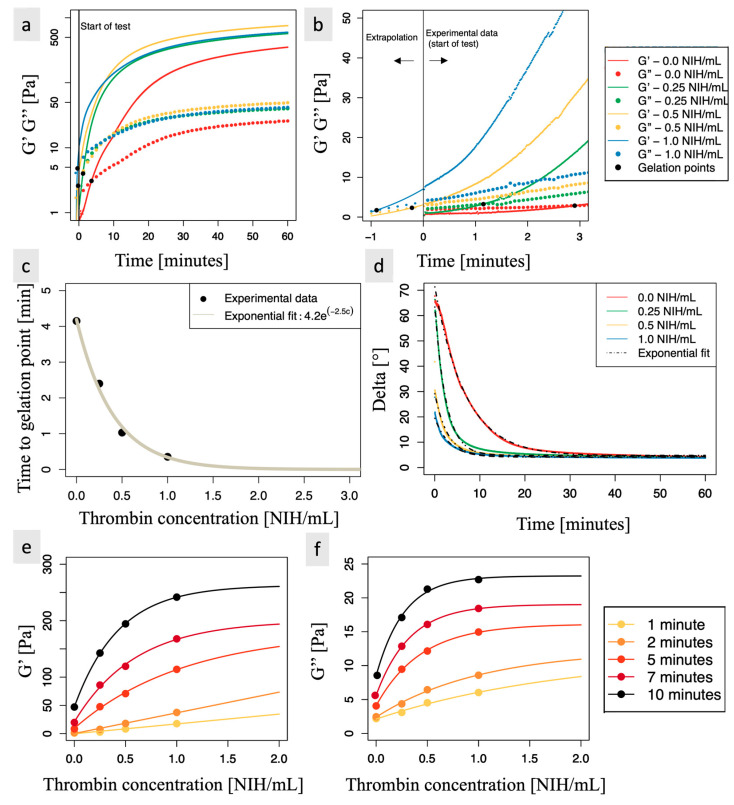
Rheological results. (**a**) Vertical black line indicates the beginning of the test (t = 0 min). On the right of this vertical line: Time sweeps mean raw data for storage (G′, full lines) and loss (G″, dotted lines) modulus of biomaterial with thrombin (0.0, 0.25, 0.5, 1.0 NIH/mL) for a displacement of 1% and a frequency of 5 rad/s. On the left of the vertical black line, data were extrapolated based on the first 3 min of the test for thrombin concentrations of 0.5 and 1.0 NIH/mL to determine their gelation point. The equation $y=A+Be^{Rt}$ was used for the fit, where R≥0, y is either the loss or the storage modulus, and t is the time. Black dots indicate the gelation point (G′ = G″) for each thrombin concentration. (**b**) Zoom for t = −1 to 3 min. (**c**) Black dots show the time needed to reach the gelation point for biomaterials with each thrombin concentration (0.0, 0.25, 0.5, and 1.0 NIH/mL). In order to account for the time elapsed between the rehydration of the biomaterial and the start of the test, 1.25 min were added to the experimental time observed in panel b. The experimental data were fitted using the equation $y=Ae^{Rx}$, where R≤0 (solid curves), *y* is the time to gelation and *x* is the thrombin concentration. (**d**) Results of phase angle (δ°) for biomaterials (0.0, 0.25, 0.5, or 1.0 NIH/mL of thrombin) over 60 min. δ = $\tan^{-1}(G\prime \prime /G’)$. The experimental data was fitted using the equation $\delta =A+Be^{Rx}$, where R≤0 (solid curves), and *x* is the thrombin concentration. (**e**) Values of G′ (Pa) and (**f**) values of G″ (Pa) for biomaterials with or without thrombin (0.0, 0.25, 0.5, 1.0 NIH/mL) at 1, 2, 5, 7, and 10 min (• are experimental data). The values of G′ and G″ (Pa) at those times were empirically fitted with the equation $y=A-Be^{Rx},\;\mathrm{where}\;R\leq 0$ (solid curves), and x is the thrombin concentration. For results presented in (**a**,**d**), *N* = 2 and *n* = 2. For results presented in (**b**,**c**,**e**,**f**), *N* = 5 and *n* = 2.

Biomaterial samples containing 0, 0.25, 0.5, and 1.0 NIH/mL of thrombin were studied through rheometry. The raw results for storage (G′) and loss (G″) moduli are presented in Figure 3a. It was observed that after 60 min, values for G′ and G″ were not yet stabilized (Figure 3a has a semi-logarithmic scale), indicating that the coagulation process has not been completed for any of the samples.

The gelation point (cross-over between G′ and G″) occurred on average at 4.15 min after rehydration of FD samples in PRP for the controls and after 2.40, 1.03, and 0.35 min for the formulation containing, respectively, 0.25, 0.5, and 1.0 NIH/mL of thrombin. Of note, for 0.5 NIH/mL and 1.0 NIH/mL, this gelation point occurred before the start of the test, as it took about 75 s to start the rheology experiment after rehydration of the FD samples in PRP. The mixture was therefore already in a viscous solid state (G′ > G″) when the test was started. The time to gelation for these formulations was determined by extrapolating the raw data (G′ and G″ as a function of time), as shown in Figure 3b. For the fit, an exponential function of the form $y=A+Be^{Rt}$ was used over the first 3 min of the test, where R≥0, *y* is either the loss or the storage modulus and *t* is the time. 

The time to gelation decreased significantly with thrombin concentration and followed a negative exponential, as shown in Figure 3c. For example, a thrombin concentration of 0.5 NIH/mL allowed for a reduction of the time to gelation point by a factor of 4 compared to samples without thrombin, with a time of 1.03 min versus 4.14 min). Furthermore, the negative exponential extrapolation of the data suggests that increasing the thrombin concentration beyond 1.0 NIH/mL would bring only a limited decrease in the time to the gelation point. 

Due to the visco-elastic nature of the samples, there is a time lag between the movement of the rheometer and the response signal. This time lag, δ, was calculated using the formula $\tan^{-1}\left(G\prime \prime /G\prime \right)$. A δ value closer to 90° indicates a mainly viscous behavior, as seen in the formulation containing 0 and 0.25 NIH/mL of thrombin at the beginning of the test. Conversely, a δ value closer to 0° indicates mainly elastic behavior. When modeled as a negative exponential, δ reached a constant value of approximately 4.5° for all thrombin concentrations used, as shown in Figure 3d. This indicates that the coagulated material was in an elastic solid state, as expected. Thrombin-containing biomaterials reached this near equilibrium faster than controls. 

To model the relationship between thrombin concentration and each modulus at a given time, a model of the form $y=A-Be^{Rx}$, where R≤0 and *x* is the thrombin concentration, was empirically chosen. A represents the maximal modulus value at a given time (the asymptotic value obtained with an infinite thrombin concentration), A−B is the modulus value at a given time when no thrombin is added to the biomaterial (as thrombin is naturally present in the blood, the biomaterial is still able to coagulate, so (A−B) > 0 and increases with time).

Here, the values of G′ and G” characterize the coagulation “state”. Within the first 5 min of coagulation, increasing thrombin concentration had an almost linear impact on the coagulation state at a given time, as observed in Figure 3e for G′ and in Figure 3f for G″. However, after 5 min, the effect of increasing thrombin concentration on the coagulation state was saturated for high thrombin concentrations. Based on the chosen exponential model, after 5 min, the 0.5 NIH/mL sample had already reached 40% of the maximal G′ value and 76% of the maximal G″ value, where the maximal values were extrapolated using an infinite thrombin concentration. In comparison, after 5 min, the control only reached 5% of the maximal G′ value and 25% of the maximal G″ value, while the 1.0 NIH/mL samples reached 63% of the maximal G′ value and 93% of the maximal G″ value. All these percentages increased with time, as expected. It should be noted here that the 0.5 NIH/mL samples allowed a significant increase in G′ and G″ compared to controls and 0.25 NIH/mL samples while still being easy to manipulate, as discussed in Section 3.2. In contrast, the 1.0 NIH/mL samples allowed an even more significant increase in storage and loss modulus but were less easy to manipulate. Using the exponential model, the percentages of maximal G′ or G″ values reached after 5, 7, and 10 min for all thrombin concentrations tested are presented in Table 1. 

### 3.4. Indentation Experiments Indicate That There Is No Significant Difference in Stiffness and Equilibrium Force between the Coagulated Biomaterials Containing Thrombin and the Control Samples

**Figure 4:**
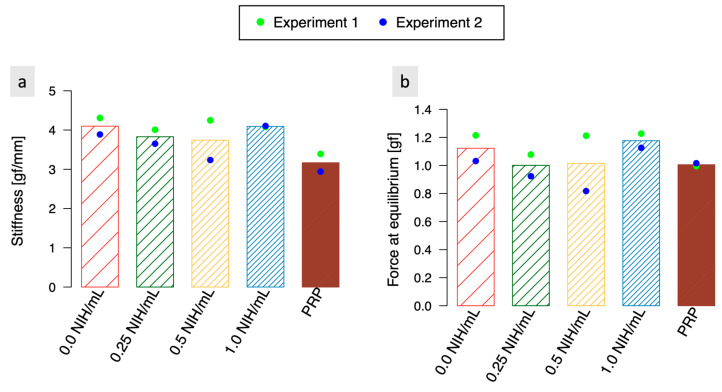
(**a**) Average stiffness and (**b**) force at equilibrium in response to an indentation compressive strain (amplitude of 0.7 mm—10% of sample’s height—speed of 0.07 mm/s) for biomaterials with different concentrations of thrombin (0.0, 0.25, 0.5, and 0.1 NIH/mL) as well as for PRP. The presence of additional thrombin appears to leave the mechanical properties of the completely coagulated clot unchanged. Both the stiffness and the force at equilibrium remained stable across every thrombin concentration tested. A difference is, however, noticeable between the stiffness of the biomaterial and the stiffness of PRP, indicating that the presence of CS increases the resistance of the biomaterial to compression. CS does not appear to have an impact on the equilibrium force. *N* = 2, and *n* = 3.

Despite its ability to increase the speed of the coagulation process, additional thrombin does not seem to have a significant impact on the mechanical properties of the fully coagulated clot, as the force at equilibrium and stiffness were stable regardless of the thrombin concentration tested, and no correlation was observed between either of them and thrombin concentration (Figure 4). The average stiffness varied only slightly from 3.7 gf/mm (material with 0.5 NIH/mL of thrombin) to 4.1 gf/mm (material without thrombin), as shown in Figure 4a, and the averaged equilibrium force varied only slightly from 1.00 gf (material with 0.25 NIH/mL of thrombin) to 1.18 gf (material with 1.0 NIH/mL of thrombin), as shown in Figure 4b. A difference is, however, noticeable between the stiffness of the biomaterial (mean of 3.9 gf/mm across all 4 concentrations) and the stiffness of PRP (mean of 3.1 gf/mm), indicating that the presence of CS increases the resistance of the biomaterial to compression. CS does not appear to have an impact on the equilibrium force. 

### 3.5. The Adherence of the Biomaterial to Tendon Tissues Is Impacted by the Biomaterial-Tendon Contact Duration and Increases Faster When Thrombin Is Present

**Figure 5:**
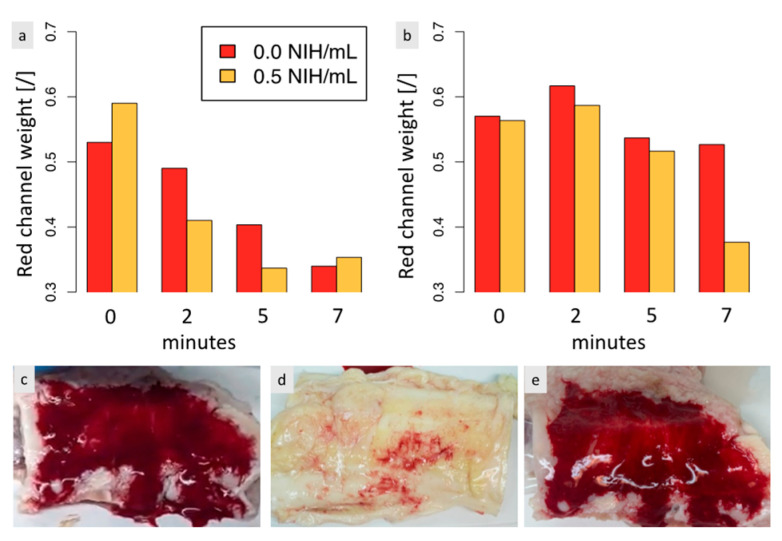
Quantification of the adherence of the biomaterial to tendon tissues when soaked in 0.9% NaCl for biomaterial with and without 0.5 NIH/mL of thrombin. After soaking, a photo of the NaCl solution was taken, and the amount of biomaterial detached from the tendon was quantified by its red channel weight. (**a**) The biomaterial was injected into the tendon immediately after reconstitution with PRP and left for 0, 2, 5, or 7 min. The tendon was then soaked in 0.9% NaCl. (**b**) The biomaterial was left for 0, 2, 5, or 7 min in a 10-cc syringe before being placed on the tendon. A 2 min wait was then observed before soaking the tendon in 0.9% NaCl. (**c**) Aspect of the biomaterial just after its injection on the tendon, before soaking. (**d**) When no waiting time is observed after injection of the biomaterial, the biomaterial almost completely dissolves in 0.9% NaCl when soaked. (**e**) When a 5-min waiting time is observed after injection of the biomaterial containing 0.5 NIH/mL of thrombin, the biomaterial does not dissolve when soaked in 0.9% NaCl, and its aspect on the tendon seems unchanged. For results presented in (**a**,**b**), *N* = 2 and *n* = 2.

Ex vivo studies were performed to investigate the adherence between the biomaterial and tendon tissues, examining the impact of the biomaterial-tendon contact duration and the biomaterial incubation time in the syringe before being delivered to the tendon. We wanted to study (1) if the adherence increased with the contact time and (2) if the adherence decreased if the coagulation process had already evolved for a few minutes in the syringe. Results indicated that the adherence of the biomaterial to tendon tissues was significantly affected by the contact duration, with a longer waiting time resulting in less biomaterial dissolution in the fluid, as shown in Figure 5a. The adherence increased faster with the presence of thrombin, which accelerated the coagulation process. In contrast, our results indicate that the time the biomaterial spends in the syringe before application has less impact on adhesion than the biomaterial-tendon contact duration. Moreover, increasing the incubation time in the syringe did not reduce adhesion but instead enhanced it, as shown in Figure 5b. As a result, surgeons can safely wait a few minutes after rehydrating and placing the biomaterial in the syringe before injecting it.

Figure 5c–e show the biomaterial on the tendon immediately after injection, after a 1-min plunge in fluid following its application, and after a 1-min plunge in fluid following a 5-min wait after its application, respectively. Most of the biomaterial dissolved in the fluid after 1 min in the absence of a waiting period, while most of it remained on the tendon after a 5-min waiting period.

### 3.6. Limitations

One limitation of this study is the use of sheep blood to investigate a biomaterial that will eventually be prepared with human PRP and used in humans. Although sheep’s blood was found to be more similar to human blood than pig, rabbit, or dog blood, significant differences in coagulation exist between these blood types [36]. In humans, the onset of coagulation occurs later than in sheep, and the coagulation process is slower [36]. Furthermore, human thrombin was used with sheep blood, and it is important to note that thrombin from one species may not produce the same results in another species as interferences may occur in the clotting reaction [37,38,39]. Given the above, we anticipate that the incorporation of human thrombin into our biomaterial will have a more pronounced effect when used in conjunction with human PRP. Currently, in the clinical trials, the biomaterial must rest in a syringe for 30–45 min after rehydration to obtain the desired consistency, which can be challenging and time-consuming during surgical procedures. Nevertheless, our results strongly suggest that the inclusion of thrombin will resolve this issue and facilitate the use of our biomaterial in clinical settings. 

## 4. Conclusions

This proof-of-concept study successfully validated our two initial hypotheses. Firstly, it demonstrated that thrombin has the potential to accelerate the solidification of CS-PRP FD implants. Secondly, thrombin can be added to the formulation before FD, and a balance can be achieved to obtain a biomaterial that is both fast to coagulate and easy to handle/inject. Moving forward, it is important to conduct animal studies to provide further evidence that CS-FIIa-PRP implants are safe and possess the same regenerative potential as CS-PRP implants. Additionally, future in vitro studies using the methodology developed here will allow us to investigate the solidification of CS-FIIa biomaterial rehydrated in human PRP and determine the optimal thrombin concentration. We are also conducting ongoing stability studies at −20 °C, 4 °C, and RT to further evaluate the performance of our biomaterial. With continued research and evaluation, we are confident that our CS-FIIa-PRP biomaterial has great potential for use in regenerative medicine applications.

## Figures and Tables

**Table 1:** Percentage of maximal G′ or G″ value reached after a given time for a given thrombin concentration.

Time [min]	0.0 NIH/mL		0.25 NIH/mL		0.5 NIH/mL		1.0 NIH/mL	
	G′	G″	G′	G″	G′	G″	G′	G″
5	5%	25%	25%	58%	40%	76%	63%	93%
7	10%	30%	41%	67%	61%	85%	83%	97%
10	18%	37%	54%	75%	74%	90%	92%	98%

## Data Availability

The data presented in this study are available on request from M.L. (the corresponding author). The data are not publicly available due to privacy restrictions.

## Supplementary Materials

The following supporting information can be downloaded at: https://zenodo.org/record/7992063. Video S1 shows the coagulation of the biomaterial for the analyzed thrombin concentrations.

## Appendix A. Assessment of Clotting Properties of CS-PRP Formulations through Rheology Measurements

The linear viscoelastic region of the samples was observed to be from 0 to 2% strain, which is in accordance with previous studies on blood coagulation [40,41]. A fixed 1% strain was therefore chosen for time sweeps tests. To determine the frequency to use in time sweeps, a frequency sweep was performed. The sample appeared as a viscous solid at high frequencies (>40 rad/s) before appearing as a viscoelastic liquid at lower frequencies. This can be indicative of the sample’s behavior at high frequencies: it was previously reported for non-coagulated blood and other macromolecular solutions that G′ (storage modulus) > G′ (loss modulus) for high frequencies (>10 rad/s) [42,43]. A hypothesis given for this behavior is that for low frequencies, the system predominantly loses energy since the translational movement of molecules through the solvent predominates over contortion. For higher frequencies, the movements of chain segments become more important, and G′ increases. This increase in G′ can be attributed to the mechanism for storing elastic energy, which is probably the contortion of plasma proteins and the deformation of individual RBCs. This hypothesis is given by Alves et al. [42]. For safety measures, a fixed frequency of 5 rad/s was therefore used in time sweeps tests. In light of those results, rheological properties were measured during a time sweep over the course of either 1 h or 15 min at a fixed 1% strain and a fixed 5 rad/s frequency. All measurements were performed at 37 °C.
